# Effects of Ti and Cu Addition on Inclusion Modification and Corrosion Behavior in Simulated Coarse-Grained Heat-Affected Zone of Low-Alloy Steels

**DOI:** 10.3390/ma14040791

**Published:** 2021-02-07

**Authors:** Yuhang Wang, Xian Zhang, Wenzhui Wei, Xiangliang Wan, Jing Liu, Kaiming Wu

**Affiliations:** The State Key Laboratory of Refractories and Metallurgy, Hubei Province Key Laboratory of Systems Science in Metallurgical Process, Collaborative Innovation Center for Advanced Steels, Wuhan University of Science and Technology, Wuhan 430081, China; wangyuhang@wust.edu.cn (Y.W.); weiwenzhui@126.com (W.W.); wanxiangliang@wust.edu.cn (X.W.); liujing2015@wust.edu.cn (J.L.)

**Keywords:** low-alloy steel, SEM, inclusion, anodic dissolution, pitting corrosion

## Abstract

In this paper, the effects of Ti and Cu addition on inclusion modification and corrosion behavior in the simulated coarse-grained heat-affected zone (CGHAZ) of low-alloy steels were investigated by using in-situ scanning vibration electrode technique (SVET), scanning electron microscope/energy-dispersive X-ray spectroscopy (SEM/EDS), and electrochemical workstation. The results demonstrated that the complex inclusions formed in Cu-bearing steel were (Ti, Al, Mn)-O_x_-MnS, which was similar to that in base steel. Hence, localized corrosion was initiated by the dissolution of MnS. However, the main inclusions in Ti-bearing steels were modified into TiN-Al_2_O_3_/TiN, and the localized corrosion was initiated by the dissolution of high deformation region at inclusion/matrix interface. With increased interface density of inclusions in steels, the corrosion rate increased in the following order: Base steel ≈ Cu-bearing steel < Ti-bearing steel. Owing to the existence of Cu-enriched rust layer, the Cu-bearing steel shows a similar corrosion resistance with base steel.

## 1. Introduction

Low-alloy steel has been widely used as a construction material in the marine environment, owing to its remarkable mechanical properties and low cost [1,2,3]. In the harsh marine environment, low-alloy steels are susceptible to localized corrosion due to the existence of aggressive anions, such as Cl^−^ and SO_4_^2−^ [4,5,6]. Moreover, localized corrosion induced by inclusions is usually in conjunction with a high local corrosion rate, which can result in a structural failure [7].

The weldability of low-alloy steel had a significant impact on its application. In the construction of bridges, ships, and steel structures, high heat input welding was used to improve welding productivity. However, the grain coarsening appeared in the heat-affected zone during the welding process, leading to degraded mechanical properties [8]. Moreover, residual stress generated during the solidification and shrinkage process can facilitate the formation of cold cracking in the heat-affected zone [9,10]. To improve the mechanical property of the coarse-grained heat-affected zone (CGHAZ) induced by high heat input welding, alloying elements, such as Ti and Cu, were added to refine the microstructure during the welding process [11,12,13]. The addition of Ti generates the dispersive and fine TiN precipitates, which effectively hinders the migration of grain boundaries. Thus, the addition of a small amount of Ti significantly inhibited the prior austenite grain growth [14,15]. It has been reported [16,17] that Cu addition effectively improves the acicular ferrite fraction, which would lead to a superior impact toughness.

However, the addition of alloying elements can significantly affect the corrosion resistance of the steel, owing to its influence on protective rust layer formation, inclusion number density, size distribution, and chemical composition [18,19,20]. In Cu and Cr containing steel, an elemental Cu- and Cr-enriched layer would generate on the steel surface, this compact rust layer can significantly inhibit both anodic and cathodic reactions, lowering the corrosion rate of steel [18,19,21]. With the addition of alloying elements, such as S and Al, the number density and average size of MnS, MnO, and Al_2_O_3_ obviously increased, which would lead to the decrease of corrosion resistance [20,22,23]. On the contrary, the addition of RE elements can improve the corrosion resistance of the steel, owing to the much smaller average size of the modified inclusions than that of normal inclusions [24,25,26,27].

In our previous work, the mechanism of pitting initiation and propagation process induced by (Zr-Ti-Al)-O_x_ inclusions in Zr-Ti deoxidized low-alloy steel were thoroughly investigated [28]. In previous studies, the impact of Ti and Cu addition on microstructure and toughness in simulated CGHAZ of low-alloy steels [15,16] and the localized corrosion behavior induced by inclusions, such as Al_2_O_3_, MnS, were discussed in detail [29,30,31]. However, with addition of Ti, Cu, and Mn, the correlation between inclusion modification and corrosion behavior in simulated CGHAZ of low-alloy steel has not yet been established.

In the present work, the effect of Ti and Cu addition on inclusion modification and corrosion behavior in CGHAZ of low-alloy steel was investigated. First, the inclusion number density, size distribution, and chemical composition with the addition of Ti and Cu were characterized by field-emission scanning electron microscopy/energy-dispersive spectrometry (FE-SEM-EDS). In addition, an immersion test coupled with in situ scanning vibrating electrode technique (SVET) and SEM/EDS was used to investigate the pit initiation and the propagation process induced by inclusions. Moreover, potentiodynamic polarization measurement was employed to analyze the corrosion resistance in the CGHAZ of Ti, Cu addition, and base low-alloy steels. Finally, the impact of inclusion number density, average diameter, and chemical composition on corrosion behavior was clarified.

## 2. Materials and Experimental

### 2.1. Sample Preparation

Three experimental steels, micro-alloyed with different Cu and Ti content, were prepared in a 10 kg vacuum melt induction furnace (Wuhan University of Science and Technology, Hubei, China), indicated as base (X70 pipeline) steel, Cu-bearing steel, and Ti-bearing steel, respectively. The chemical compositions of the investigated steel are listed in Table 1. The cylindrical ingots with 120 mm in diameter and 100 mm in length were reheated to 1250 ± 20 °C and forged into the cuboid with cross-section of 30 mm × 30 mm. A thermal simulator (Gleeble 3800, DSI, Bolingbrook, IL, USA) was used to simulate the CGHAZ. The samples were machined into cuboid with the dimensions of 11 mm × 11 mm × 100 mm. To simulate the submerged-arc welding at heat input of 100 kJ·cm^−1^, the thermal cycle simulation was proceeded with a peak temperature of 1350 °C, a heating rate of 300 Ks^−1^, and a holding time of 3 s. Besides, the cooling time from 800 to 500 °C was 52.8 s [16]. The heat input and the cooling time from 800 to 500 °C satisfy the Equation (1):

(1) $$t=\left(0.67-5\times 10^{-4}T_{0}\right)\;\times E\times \left(\frac{1}{500-T_{0}}-\frac{1}{800-T_{0}}\right)$$

where *T*_0_ is the initial temperature 20 °C. Then, it can be obtained that the cooling times from 800 °C to 500 °C was 52.8 s, which were approximately equivalent to welding at the heat input of 100 kJ·cm^−1^. The absorbed energy in the simulated CGHAZ of these three types of steels at −20 °C was measured by Charpy V-notch test (Table 2). Samples cut from the simulated CGHAZ with a size of 10 mm × 5 mm × 5 mm were sequentially ground with 2000 grit SiC paper and then polished with diamond paste down to 2.5 μm, ultrasonically degreased in ethanol.

### 2.2. Surface Characterization

The prior austenite grain diameters of three types of steels were calculated by Nano Measurer, and about nine optical micrographs with 200 magnification were measured for each sample. The number density, size distribution, and chemical composition of inclusions were measured by an SEM (EVO MA10, ZEISS, Oberkochen, Germany). The morphology and elemental distribution of the inclusions were observed by an FE-SEM (Apreo S HiVac, FEI, Hillsboro, OR, USA) equipped with an EDS (AZteclive Ultim Max 100, OXFORD Instruments, Abingdon, UK).

To observe the localized corrosion process induced by inclusions of these steel, immersion tests (10 min) were implemented in 0.5 wt.% NaCl solution. After the immersion test, approximately ten inclusions in these samples were characterized via SEM/EDS to obtain the corrosion morphology and elemental distribution, which can be used to analyze the mechanism of localized corrosion induced by inclusions in CGHAZ.

### 2.3. In Situ Scanning Vibration Electrode Technique

A SVET system (Versascan, Ametek, Berwyn, PA, USA) was used to conduct the in situ micro-electrochemical measurement, which can produce the distribution and magnitude of local current intensities on the corroding surface of the samples in 0.5% NaCl solution. On the corroding surface of the steel, oxidation and reduction reactions usually occurred in separate regions. Owing to the nature of the difference of each reaction, an electric field was generated in the solution [32]. These extremely small potential variations over the corroding surface could be detected by the scanning vibration probe. For the test, the vibrating electrode was an insulated Pt-Ir probe with a diameter of approximately 5 μm, and its vibration frequency was 80 Hz and a vibration amplitude of 30 μm. According to the noise frequencies, the lock-in amplifier could filter out the electrical noise and then transformed the potential vibration into local current density by Ohm’s law: I = −ΔE/R, where R = d/k, d represent the amplitude of the microelectrode (30 μm), and k represent the conductivity of the solution (8.42 mS·cm^−1^ for 0.5% NaCl solution) [23].

### 2.4. Electrochemical Tests

The potentiodynamic polarization tests were conducted in 0.5% NaCl solution at 25 ± 1 °C via an electrochemical workstation (E4, Zahner, Kronach, Germany). During the tests, a three-electrode cell was composed by a counter electrode of platinum plate, a saturated calomel (SCE) reference electrode and the steel sample as the working electrode. The scan range of potentiodynamic polarization tests was from −300 mV_vs.OCP_ to 300 mV_vs.OCP_ and a scan rate of 0.5 mV·s^−1^. The potentiodynamic polarization tests for each sample were conducted at least three times for reproducibility.

## 3. Results

### 3.1. Microstructure Characterization

**Figure 1:**
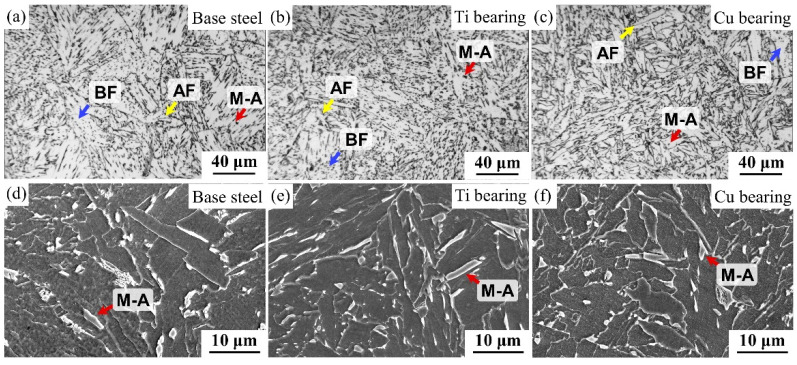
Optical micrographs and SEM micrographs in the simulated CGHAZ of three types of steels. BF, Bainite ferrite; AF, Acicular ferrite; (**a**) Optical micrographs of base steel; (**b**) Optical micrographs of Ti bearing steel; (**c**) Optical micrographs of Cu bearing steel; (**d**) SEM micrographs of base steel; (**e**) SEM micrographs of Ti bearing steel; (**f**) SEM micrographs of Cu bearing steel.

The micrographs of the simulated CGHAZ of the three types of steels after thermal cycles are shown in Figure 1. In the simulated CGHAZ, the prior austenite grain diameter of base steel, Ti-bearing steel, and Cu-bearing steel was 66, 61, and 60 μm, respectively. The microstructures in the simulated CGHAZ of the three types of steels were composed of bainite, acicular ferrite, and martensite-austenite (M-A) constituents (Figure 1). The fractions and distribution of the M-A constituents and acicular ferrite in these three types of steels were slightly different, which was thoroughly investigated in previous studies [15,16]. The focus of this work is the influence of element addition on the formation of inclusions and corrosion behavior of the steels.

### 3.2. Characterization of Inclusions

**Figure 2:**
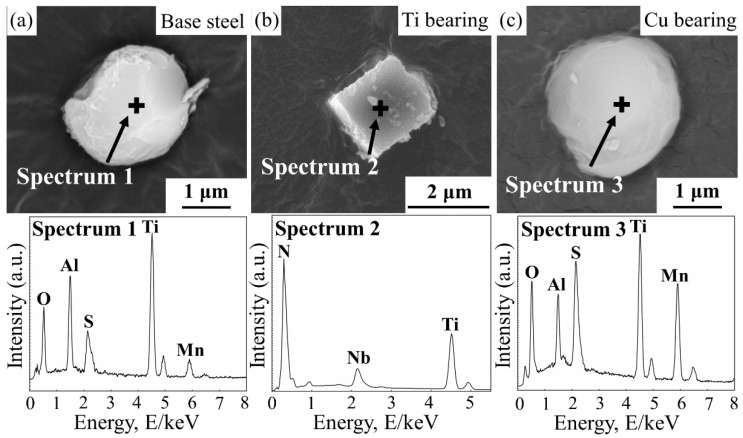
SEM images and corresponding EDS results of extracted inclusions in three types of steels. (**a**) Base steel. (**b**) Ti-bearing steel. (**c**) Cu-bearing steel.

The elemental composition, number density, and average diameter of the complex inclusions in CGHAZ of three types of steels were statistically analyzed via an SEM/EDS, and the result is shown in Table 3. In base steel, the main types of inclusions were MnS, (Ti, Al)-O_x_ and (Ti, Al, Mn)-O_x_-S_y_. In Ti-bearing steel, the main types of inclusions were Al_2_O_3_, TiN and TiN-Al_2_O_3_. In Cu-bearing steel, the main types of inclusions were MnS, (Ti, Al, Mn)-O_x_ and (Ti, Al, Mn)-O_x_-S_y_. Figure 2 shows the morphologies and chemical composition of extracted inclusions with the highest number density in three types of steels. Different from the globular feature of inclusions in base steel and Cu-bearing steel, the shape of inclusions in Ti-bearing steel was obviously irregular and acute.

**Figure 3:**
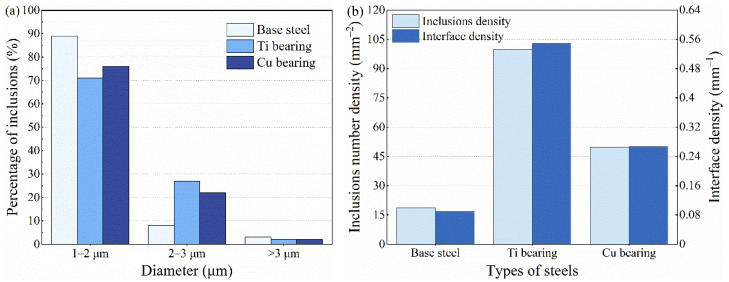
(**a**) The diameter distribution of inclusions, (**b**) number density and interface density of inclusions.

The ability of inclusions to induce localized corrosion was not only limited by the chemical composition but also influenced by the dimension and density of inclusions [25,27,33,34]. Hence, the number density and size distribution of inclusions in three types of low-alloy steels were statistically analyzed via an SEM/EDS (Figure 3a,b). With the increased content of Ti and Cu elements, the density of inclusions obviously increased in two types of steels (Figure 3b). The number density of inclusions in Ti-bearing steel and Cu-bearing steel increased to 5.4 and 2.7 times that in base steel, respectively (Figure 3b). Additionally, the fractions of coarse inclusions (2–3 μm) in Ti- and Cu-bearing steels were 29% and 24%, respectively, which significantly increased in comparison with the base steel (11%) (Figure 3a).

Owing to the micro-crevices and high lattice distortion regions around the inclusions, the initiation site of localized corrosion was generally located in the steel matrix/inclusion interface [26,35,36,37,38]. Therefore, the length of the interface between inclusions and the steel matrix per unit area was defined as interface density *ρ_i_*, which can be calculated by Equation (2) [39]:

(2) $$\rho_{i}=\pi \rho R$$

where *ρ* is the number density of the inclusions, *R* is the average diameter of the inclusions. It was noticeable that as Ti and Cu elements content increased, the interface density in Ti-bearing steel and Cu-bearing steel increased to 6.1 and 3.0 times that in base steel (Figure 3b), respectively. The interface density showed a similar trend with the number density, but it was obvious that the interface density of Ti-bearing steels grows more than number density due to the larger average diameter of inclusions, which effectively reflected the influence of inclusions average diameter on corrosion initiation. To gain more insight into the influence of element addition on corrosion behavior, the initiation and propagation process of localized corrosion were investigated by the in-situ immersion tests in the next section.

### 3.3. Immersion Test

**Figure 4:**
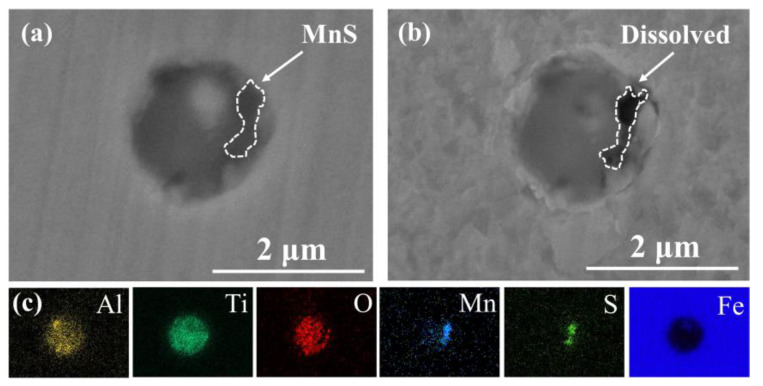
(**a**) SEM image of a typical complex inclusion in base steel. (**b**) Corrosion morphology of inclusion in (**a**). (**c**) EDS maps of inclusion in (**a**).

**Figure 5:**
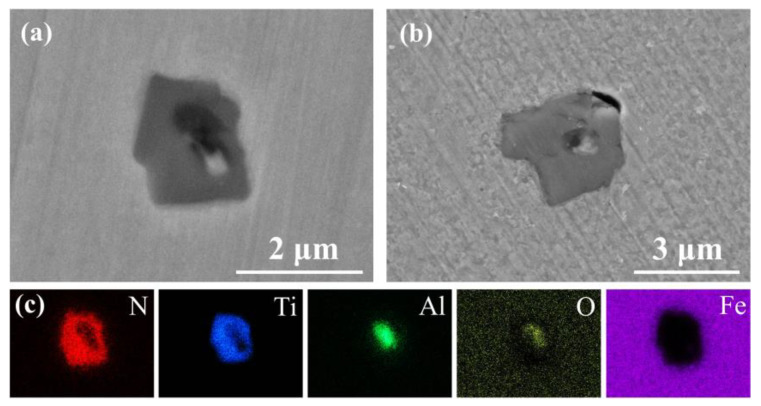
(**a**) SEM image of a typical complex inclusion in Ti-bearing steel. (**b**) Corrosion morphology of a typical inclusion. (**c**) EDS maps of inclusion in (**a**).

**Figure 6:**
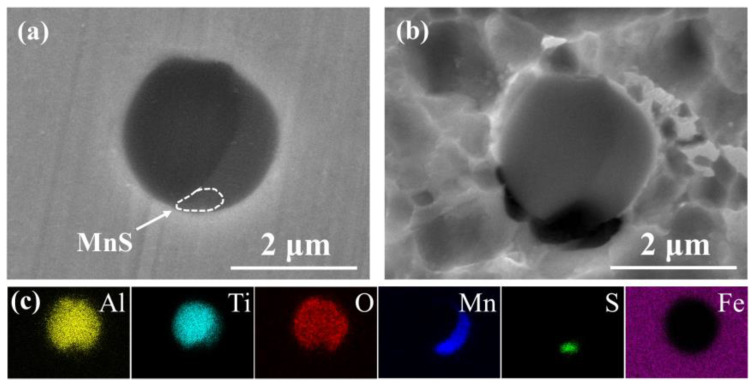
(**a**) SEM image of a typical complex inclusion in Cu-bearing steel. (**b**) Corrosion morphology of inclusion in (**a**). (**c**) EDS maps of inclusion in (**a**).

The surface morphology and elements distribution of inclusions in CGHAZ of three types of steels before and after the immersion test were characterized by means of SEM/EDS (Figure 4, Figure 5 and Figure 6).

**Figure 7:**
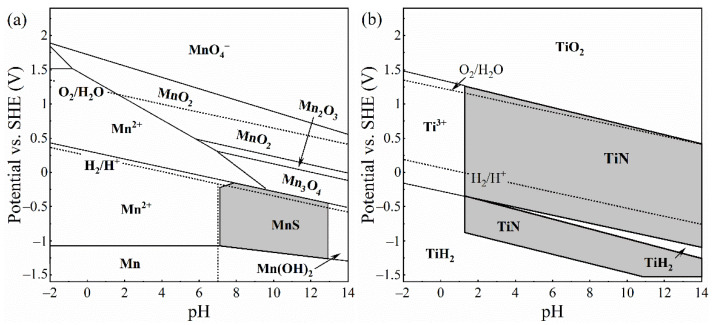
Potential-pH diagrams of MnS-H_2_O and TiN-H_2_O systems at 25 °C. (**a**) MnS-H_2_O system (Adapted with permissions from ref. [42]. 2021 Springer Nature.); (**b**) TiN-H_2_O system (Adapted with permissions from ref. [45]. 2021 Elsevier.).

The complex inclusion in the base steel was composed of two components. The oxide component in complex inclusions was (Al, Ti)-O_x_, and the sulfide component is MnS (Figure 4a,c), which is consistent with the literature [26,40,41]. After the immersion tests, the complex inclusions in the base steel partially dissolved (Figure 4b). According to the EDS results, MnS in the complex inclusion completely dissolved and the residual part of the inclusions was (Al, Ti)-O_x_ (Figure 4). As shown in the potential-pH diagram for MnS in Figure 7a [42], MnS was thermodynamically unstable in near-neutral pH solutions. Moreover, it has been reported [26] that (Al, Ti)-O_x_ owned higher electrochemical stability in comparison with the steel matrix. This indicates that the dissolution of MnS component in the complex inclusions initiated the localized corrosion [30,31,43,44].

The complex inclusion in the Ti-bearing steel was also composed of two components. The oxide component in complex inclusions was Al_2_O_3_, and the nitride component was TiN (Figure 5a,c). Besides, the Al_2_O_3_ component in the complex inclusions was surrounded by the TiN component (Figure 5a,c). After the immersion tests, a micro-crevice generated at the steel matrix/inclusion interface (Figure 5b). As can be seen in the potential-pH diagrams of TiN (Figure 7b) [45], the TiN was thermodynamically stable in the near-neutral pH solutions. Moreover, owing to the difference in thermal coefficient expansions and Young’s modulus between the steel matrix and TiN inclusions, micro-crevices and high lattice distortion regions might generate in the steel matrix/inclusion interface [29,36]. Thus, the preferential adsorption of Cl^−^ in the micro-crevices and high lattice distortion regions accelerated the dissolution of the steel matrix around the TiN inclusions, which initiated the localized corrosion during the immersion test. The composition of inclusions in the Cu-bearing steel was similar to that in the base steel. The oxide component in complex inclusions was (Al, Ti, Mn)-O_x_, and the sulfide component was MnS (Figure 6a,c). After the immersion tests, a micro-crevice was detected at the steel matrix/inclusion interface, indicating localized corrosion was induced by the dissolution of MnS component and the adjacent steel matrix (Figure 6b), which was consistent with the localized corrosion behavior of base steel.

Based on the above phenomenon, the localized corrosion behavior induced by inclusions in these three types of steels can be classified into two categories. The first category is MnS-containing complex inclusions. During the immersion test, the localized corrosion was initiated in the complex inclusions rather than the steel matrix (Figure 4 and Figure 6) owing to the lower thermodynamical stability of MnS in near neutral solution. In the second category, the Al_2_O_3_ and TiN inclusions have higher thermodynamic stability than the steel matrix. Due to the significantly nonuniform deformation between inclusion and the steel matrix during the rolling process, the high lattice distortion regions in the matrix would be the preferential dissolution site at the pit initiation stage [26,29].

### 3.4. In-Situ SVET Measurements

**Figure 8:**
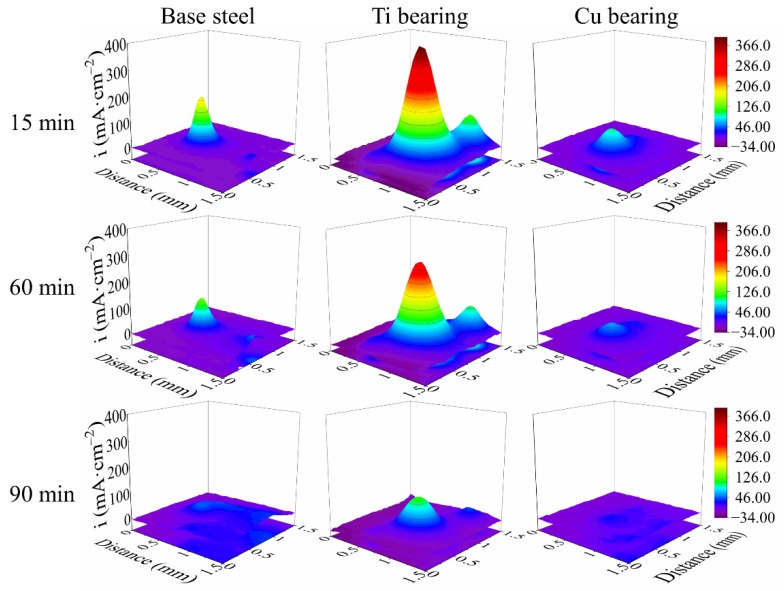
In-situ SVET maps of three types of steels in 0.5% NaCl solution.

An SVET measurement was employed to characterize the local electrochemical behavior during the localized corrosion initiation and propagation process, and the local corrosion current density maps are shown in Figure 8. During the SVET test, the local current density in anodic and cathodic sites exhibited considerable disparity (Figure 8). At the initiation stage (15 min), the localized corrosion occurred spontaneously, and the local anodic current density of the three types of steels decreased in the following order (Figure 8): Ti-bearing > Base steel > Cu-bearing. After extended exposure periods (60 min), the relatively high anodic current peak at the center of the maps gradually decreased (Figure 8). Moreover, in base steel, Cu-bearing steel samples, the anodic current peak almost disappeared at 1 h 30 min (Figure 8), which means localized corrosion gradually transformed to uniform corrosion, whereas a clear anodic current peak was observed in the Ti-bearing sample with exposure time extended to 1 h 30 min (Figure 8).

**Figure 9:**
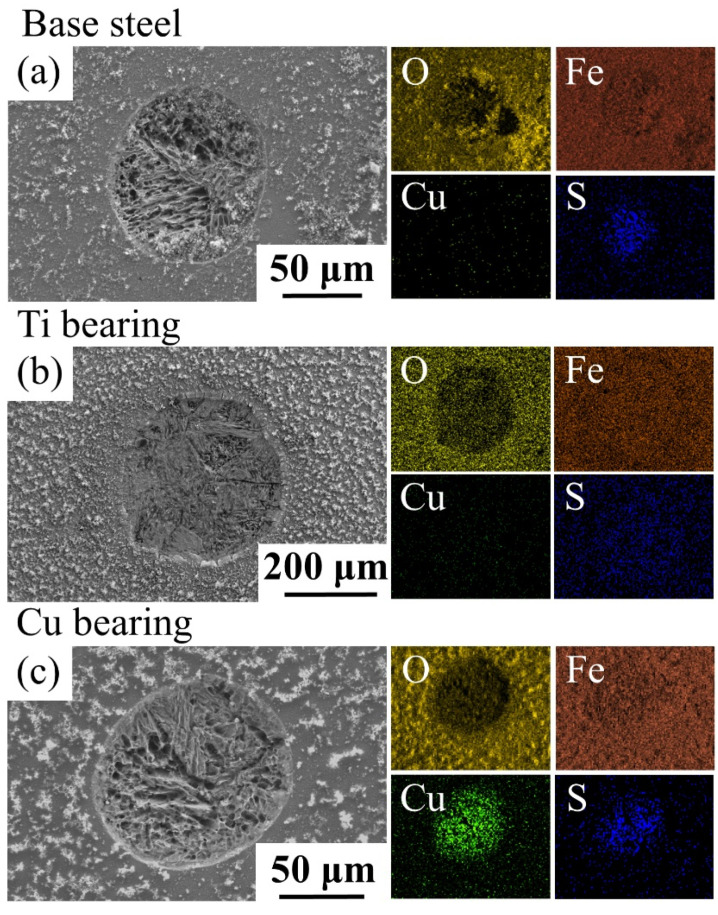
Morphology of the corrosion sites and the element distribution of the corrosion products after SVET tests. (**a**) Base steel. (**b**) Ti-bearing steel. (**c**) Cu-bearing steel.

To investigate the Cu addition on corrosion resistance of the steels, the corrosion sites after SVET tests were characterized by means of SEM/EDS. Figure 9 shows the morphologies of the corrosion sites and the elemental distribution of the corrosion products. In the anodic corrosion region of these three types of steels, the steel matrix severely dissolved, which contributed to the increase of the anodic current peak (Figure 8 and Figure 9). For these three types of samples, Fe was distributed on the whole surface, O and S were detected to be distributed on the cathodic and anodic regions (Figure 9), respectively. According to the EDS results in Figure 9, the corrosion products in the anodic region of base steel and Ti-bearing steel were iron sulfide, which is consistent with the literature [38,46]. Additionally, the accumulated iron sulfide could promote the uniform dissolution of the steel matrix in Cl^−^ containing solution [46], whereas the enrichment of Cu was observed in the anodic corrosion region of Cu-bearing sample (Figure 9c). As reported earlier, the solubilized Cu in the steel matrix can deposit on the steel surface during the steel matrix dissolution process and then enhance the compactness of the rust layers and suppress the anodic reactions [18,47]. Thus, the local corrosion current density of Cu-bearing sample was lowered by Cu-enriched rust layer (Figure 8).

### 3.5. Potentiodynamic Polarization Tests

**Figure 10:**
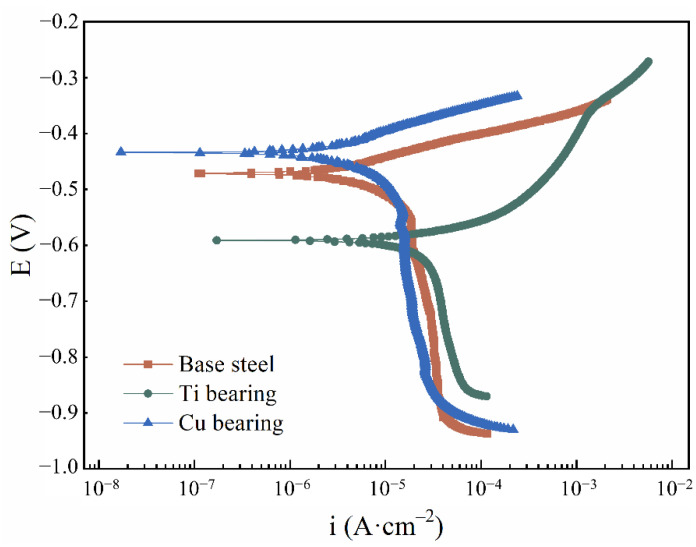
Polarization curves of three types of steels in 0.5% NaCl solution.

To evaluate the influence of element addition on corrosion resistance in CGHAZ of these three types of steels, the potentiodynamic polarization tests were carried out, and the polarization curves are displayed in Figure 10. As shown in Table 4, the electrochemical parameters were extrapolated by means of the Tafel extrapolation method, which was conducted by stretching the linear parts of the anodic and cathodic curves back to their intersection, the abscissa and ordinate of the intersection was the corrosion current density and corrosion potential, respectively [48]. As shown in Table 4, the corrosion current density increased in the following order: Base steel ≈ Cu-bearing steel < Ti-bearing steel, indicating a decreasing trend of corrosion resistance. With the increased interface density of Ti-bearing steel, the corrosion rate of steels obviously increased compared to base steel. However, Cu-bearing steel was an exception, which had higher interface density and similar corrosion rate compared to that of base steel. According to the EDS results shown in Figure 9, the superior corrosion resistance of Cu-bearing steel was attributed to the Cu-enriched rust layer covered on the surface. Additionally, owing to the different thermal expansion coefficients and Young’s modulus between inclusions and the steel matrix, the inclusion/matrix interface with micro-crevices and high lattice distortion regions was the initiation site of localized corrosion. Thus, the higher interface density obviously increased the corrosion rate of Ti-bearing steels [29,36].

## 4. Discussion

Based on the above results, it is evident that the addition of Ti and Cu modified the number density, chemical compositions, and average diameter of inclusions formed in the CGHAZ of low-alloy steels, which had a significant impact on the initiation and propagation of corrosion [49], further leading to the different corrosion resistance of steels.

**Figure 11:**
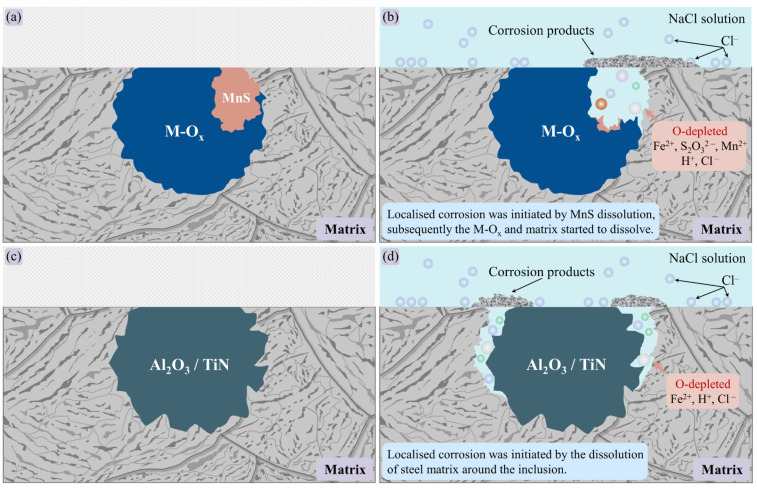
Schematic of the localized corrosion induced by inclusions. (**a**,**b**) Complex inclusions composed of M-O_x_ and MnS. (**c**,**d**) Inclusions composed of Al_2_O_3_/TiN.

The schematics of two types of localized corrosion behaviors induced by inclusions in three types of steels are shown in Figure 11. For the first types of inclusions composed of metal oxides (M-O_x_) and MnS (Figure 11a,b), the localized corrosion was initiated by the dissolution of MnS due to the thermodynamical instability of MnS in near-neutral pH solution [42]. Furthermore, the S_2_O_3_^2−^ and H^+^ were produced by the electrochemical dissolution of MnS by Equation (3) [46,50]

(3) $$2\mathrm{MnS}+3H_{2}O\to 2Mn^{2+}+S_{2}O_{3}^{2-}+6H^{+}+8e^{-}$$

which decreased the pH of solutions in the micro-crevice located in the steel matrix/inclusion interface (Figure 11b). Subsequently, the M-O_x_ and the adjacent steel matrix would be dissolved in the aggressive environment.

For the second type of inclusions, mainly composed of Al_2_O_3_/TiN (Figure 11c,d), the dissolution of the steel matrix that was distributed around the inclusions initiated the localized corrosion. Al_2_O_3_ and TiN have a much higher Young’s modulus in comparison with the steel matrix [51], thus nonuniform deformation appeared between the inclusions and the steel matrix [52]. In addition, the distinctly lower thermal coefficient expansions of Al_2_O_3_ and TiN than the steel matrix can also result in the formation of high deformation region in the steel matrix/inclusion interface [36]. It was reported [29,53,54,55,56] that the adsorption process of Cl^−^ can be significantly favored by the local deformation, generally leading to higher electrochemical activity in the deformation matrix. Hence, the steel matrix around inclusions could be easily attacked due to the existence of high deformation region, and it was the preferential dissolution site during the pit initiation process. With the steel matrix at the inclusion/matrix interface dissolved, micro-crevice formed at the interface between inclusion and matrix. The micro-crevice environment would be oxygen-depleted due to the poor convection. Consequently, cathodic reactions in micro-crevice were suppressed [27]. Therefore, the micro-crevice environment became enriched in metal cations, which resulted in the electromigration of Cl-ions into micro-crevice [57]. The pH in the micro-crevice was lowered by the cation hydrolysis

(4) $$Fe^{2+}+2H_{2}O\to \mathrm{Fe}{\left(\mathrm{OH}\right)}_{2}+2H^{+}$$

which would facilitate the propagation of localized corrosion [58].

With the dissolution of MnS or the steel matrix around the Al_2_O_3_/TiN inclusions, micro-crevice that was enriched in acid solution was formed at the inclusions/matrix interface. Subsequently, the residual inclusion and adjacent matrix dissolved owing to the existence of acid environment, and the micro-crevice was enlarged and covered by corrosion products (Figure 11b,d). Hence, a catalytic-occluded cell generated in the micro-crevice, which would considerably promote the corrosion propagation.

**Figure 12:**
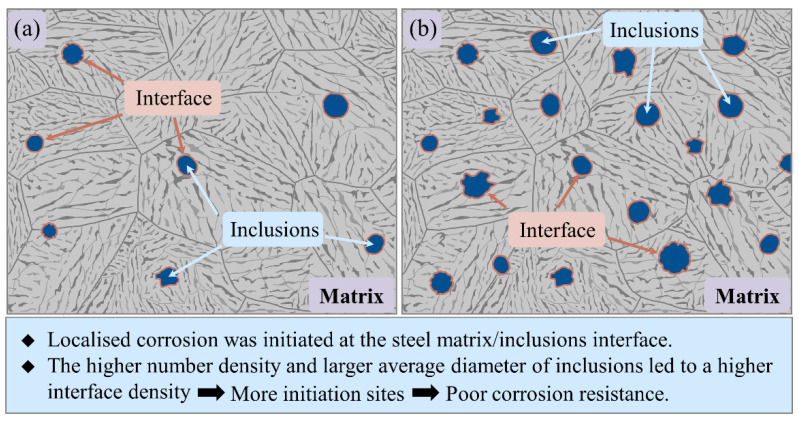
Schematic of element addition on the inclusion modification and corrosion behavior. (**a**) The steel matrix with lower number density and smaller average diameter of inclusions. (**b**) The steel matrix with higher number density and larger average diameter of inclusions.

With increased immersion time, numerous corrosion pits generated in the exposed steel surface. Owing to the interaction effects of the nearby corrosion pits, the independent corrosion pits would coalesce into larger corrosion spots [29]. Consequently, the localized corrosion would transform into uniform corrosion in the later stage. As for the uniform corrosion resistance of steel, it was affected by both the interface density of inclusions and the property of rust layers. The schematics of the interface density on corrosion resistance of the steels are displayed in Figure 12. Compared to the base steel, the higher interface density in Ti-bearing steel provided more initiation sites for localized corrosion, induced by the higher number density and the larger average diameter of inclusions. With increased localized corrosion sites in the steel surface, more iron sulfide deposited on the exposure surface around inclusions, which significantly promoted the uniform corrosion of the steel matrix. Hence, the corrosion rate of Ti-bearing steels was obviously higher than that of base steel. Although the interface density in Cu-bearing steel was higher than that in base steel, the corrosion rate of Cu-bearing steel was similar to the base steel (Table 4). According to EDS results shown in Figure 9, the solubilized Cu in the steel matrix deposited on the surface of the anodic corrosion region, which considerably suppressed the anodic reaction [18], resulting in the improvement of corrosion resistance.

## 5. Conclusions

The effects of Ti and Cu addition on the inclusion modification and corrosion behavior in simulated CGHAZ of low-alloy steels were investigated. The conclusions are summarized as follows.

(1) The number density and average diameter of inclusions in Ti-bearing steel (99.77 mm^−2^, 1.75 μm) and Cu-bearing steel (49.70 mm^−2^, 1.71 μm) were larger than in base steel (18.53 mm^−2^, 1.54 μm). The main types of inclusions in Ti-bearing steel were TiN-Al_2_O_3_, which was obviously different from that in base steel. In Cu-bearing steel, the main types of inclusions were (Ti, Al, Mn)-O_x_-MnS, which was similar to that in base steel.
(2) Localized corrosion behaviors induced by inclusions in three types of steels were categorized into two types. For the first type of inclusions, localized corrosion was initiated by the dissolution of MnS. For the second type of inclusions, the dissolution of the steel matrix around Al_2_O_3_/TiN initiated the localized corrosion due to the high deformation region in the steel matrix/inclusion interface.
(3) The corrosion rate increases in the following order: Base steel (3.91 × 10^−2^ mm·a^−1^) ≈ Cu-bearing steel (3.66 × 10^−2^ mm·a^−1^) < Ti-bearing steel (44.26 × 10^−2^ mm·a^−1^). With the increased interface density of the steels, the corrosion rate of CGHAZ in the steels obviously increased, whereas the Cu-bearing steel is an exception. The solubilized Cu in the steel matrix can enhance the compactness of the rust layers, then lower the corrosion current density.

## Figures and Tables

**Table 1:** Chemical composition of the experimental steels (wt.%). Adapted with permission from ref. [16]. 2021 Taylor & Francis.

Samples	C	Si	Mn	Nb	V	Ti	Cu	Al	Fe
Base (X70) steel	0.055	0.21	1.61	0.038	0.022	0.012	0	0.022	Balance
Ti-bearing steel	0.057	0.23	1.63	0.039	0.021	0.061	0	0.025	Balance
Cu-bearing steel	0.056	0.21	1.60	0.040	0.021	0.010	0.32	0.024	Balance

**Table 2:** Impact toughness in the simulated CGHAZ of the experimental steels.

Samples	−20 °C Absorbed Energy (J)	
	Mean	Deviation
Base steel	78	±12
Ti-bearing	6	±0.6
Cu-bearing	241	±18

**Table 3:** Statistical analysis of inclusions in three types of steels.

Sample	Types	Number Density (mm^−2^)		Average Diameter (μm)	
		Individual	Overall	Individual	Overall
Base steel	MnS	4.16	18.53	1.31	1.54
	(Ti, Al)-O_x_	4.65		1.92	
	(Ti, Al, Mn)-O_x_-S_y_	9.72		1.46	
Ti-bearing	Al_2_O_3_	5.76	99.77	1.53	1.75
	TiN	90.54		1.77	
	TiN-Al_2_O_3_	3.47		1.55	
Cu-bearing	MnS	8.27	49.70	1.39	1.71
	(Al, Ti, Mn)-O_x_	18.00		1.86	
	(Ti, Al, Mn)-O_x_-S_y_	23.43		1.70	

**Table 4:** Fitted electrochemical parameters of polarization curves in Figure 10.

Sample	i (×10^−6^ A·cm^−2^)		E (V)		CR * (×10^−2^ mm·a^−1^)	
	Mean	Deviation	Mean	Deviation	Mean	Deviation
Base steel	3.33	±0.26	–0.45	±0.017	3.91	±0.30
Ti-bearing	37.70	±3.02	–0.59	±0.037	44.26	±3.58
Cu-bearing	3.12	±0.23	–0.41	±0.016	3.66	±0.37

* CR means corrosion rate.

## Data Availability

The raw/processed data required to reproduce these findings cannot be shared at this time as the data also forms part of an ongoing study.
